# Removal of Ammonia from the Municipal Waste Treatment Effluents using Natural Minerals

**DOI:** 10.3390/molecules24203633

**Published:** 2019-10-09

**Authors:** Przemysław Seruga, Małgorzata Krzywonos, Justyna Pyżanowska, Agnieszka Urbanowska, Halina Pawlak-Kruczek, Łukasz Niedźwiecki

**Affiliations:** 1 Department of Bioprocess Engineering, Wrocław University of Economics and Business, Komandorska 118/120, 53-345 Wrocław, Poland; przemyslaw.seruga@ue.wroc.pl (P.S.); justyna.pyzanowska@gmail.com (J.P.); 2 Faculty of Environmental Engineering, Chair in Water and Wastewater Treatment Technology, Wroclaw University of Science and Technology, Wyb. Wyspiańskiego 27, 50-370 Wrocław, Poland; agnieszka.urbanowska@pwr.edu.pl; 3 Faculty of Mechanical and Power Engineering, Department of Boilers, Combustion and Energy Processes, Wroclaw University of Science and Technology, Wyb. Wyspiańskiego 27, 50-370 Wrocław, Poland; halina.pawlak@pwr.edu.pl (H.P.-K.); lukasz.niedzwiecki@pwr.edu.pl (Ł.N.)

**Keywords:** ion exchange, zeolite, bentonite, isotherm, wastewater, adsorption

## Abstract

Due to various ecological problems, it is required to remove the ammonia nitrogen from wastewater. Industrial wastewater that was not subjected to any purification was used in this study, while most processes described in the literature were carried out using synthetically prepared solutions. The study investigated the removal of ammonium ions using ion exchange on various commercial minerals, in 3 h long batch ion-exchange experiments. Furthermore, research on the sodium chloride activation of the selected mineral was conducted. The screening of the mineral with the highest removal potential was conducted taking into account the adsorption capacity (q) and maximal removal efficiency (E), based on the NH_4_^+^ ions changes determined using the selective electrode and spectrophotometric cuvette tests. The highest adsorption capacity (q = 4.92 mg/g) of ammonium ions with the maximum removal efficiency (52.3%) was obtained for bentonite, with a 0–0.05 mm particle size. After pretreatment with a 1 mol/L NaCl solution, maximum efficiency increments were observed (55.7%). The Langmuir adsorption isotherm corresponds well with the equilibrium adsorption data (R^2^ from 0.97 to 0.98), while the Freundlich model was found to be mismatched (R^2^ = 0.77). Based on these results it was concluded that natural sorbents may be effectively applied in wastewater treatment. It can be observed that as the size of sorbent particles gets lower, the adsorption capacity, as well as the removal efficiency, gets higher. The bentonite pretreatment with the NaCl solution did not result in the expected efficiency improvement. The 2 mol/L solution affected about 3.5% of the removal efficiency yield.

## 1. Introduction

Municipal, industrial, and agricultural activities generate ammonia nitrogen discharges into environmental resources. The excessive accumulation of ammonium that is discharged into water can cause serious ecological problems, such as: The accelerated eutrophication of lakes and rivers, the depletion of dissolved oxygen, and toxicity in fish and other aquatic animals in the water body [1]. The removal of ammonia from processes or waste effluents is required, due to its toxicity.

The effluent treatment processes are based on the elimination of pollutants (anaerobic and aerobic biological processes, adsorption, chemical oxidation, or combustion) [2,3] or concentration (flocculation, precipitation, ultrafiltration, nanofiltration, reverse osmosis, and evaporation) [4,5].

The most widely used methods for removing ammonia from wastewater are air stripping [6,7], ion exchange [8,9], breakpoint chlorination [10], and biological nitrification-denitrification [11,12]. The traditional method of removing ammonia from municipal and industrial wastewaters is based on biological treatments. However, it can be easily inhibited by toxic shock, pH change, low-dissolved oxygen, and low temperature in winter [11,12].

Removal of the nitrogen load through the precipitation of struvite (MgNH_4_PO_4_ × 6H_2_O) is another applicable method for wastewater treatment. However, it is limited by several factors, such as: pH, temperature, as well as magnesium, phosphorus, and calcium content. In the case of a high content of NH_4_^+^ ions, the addition of Mg^2 +^ or PO_4_^3-^ and maintenance of a weakly alkaline environment is required to achieve the crystallization of struvite [13].

Natural zeolites are porous materials, which have a three-dimensional framework structure with cavities. Their physico-chemical properties include high cation exchange capacity, cation selectivity, and high void volume [14,15]. Thus, they have many industrial applications, ranging from being used in food supplements or medical devices, to environmental remediation [16], including wastewater treatment. The factors that have an impact on the removal of ammonium from the effluents are mainly pH, temperature, reaction time, particle size, initial concentration of ions, and adsorbent dosage [17].

The ion exchange (IE) by the means of natural minerals, such as zeolites or bentonites, became more competitive and interesting because of its low cost, and its relative simplicity of application and utilization. Furthermore, IE enables the recovery of the ammonium and its reuse for other purposes, e.g., as N-fertilizer [2]. The usage of natural sorbents in N recovery can be helpful in modification of the natural N cycle, to avoid further growth of anthropogenic reactive N environmental impact and its changes [18]. Thus, it is important to foster different wastewater treatment methods other than the biological nitrification-denitrification, which only removes yet does not recover ammonium. Zeolite fully corresponds with this idea, as it is beneficial to soil and crops as a supplementary material to common fertilizers [19], as it increases the nitrification rates of the applied NH_4_^+^ to soil, and, consequently, the N-uptake in plants [20].

Although natural minerals possess advantages such as good selectivity to NH_4_^+^, good availability, and low cost, they have not been widely used on a commercial scale for wastewater treatment, probably because the exchanged minerals require further disposal, or because of the application of the regeneration process [15].

The results, reported in various works, focused on the factors that impact ammonium removal, however, these works were based on the adsorption with particular minerals and synthetic ammonia solutions [14,15], while this study uses the industrial wastewater collected from mechanical-biological treatment (MBT) plants. Furthermore, no purification of the effluent was carried out before the IE trials, and research was performed in situ, directly in the plant. In addition to that, a review of the published literature indicates that there is a significant variation in ion exchange characteristics and structure regarding zeolites from different sources, as well as suspensions found in wastewater, that also hinder the treatment process. Moreover, in general, each study was performed using a single mineral, while in this study a comparison of different sorbents applied to the same effluent was conducted.

The main aim of this study was to examine the possibilities of removing the ammonium ions from the industrial effluents using various commercial minerals. An investigation and comparison of the NH_4_^+^ ion removal was carried out. Furthermore, the study on the sodium activation of the chosen mineral was conducted. Based on the research, the novel method for wastewater treatment that was originally applied in the MBT plant was determined.

## 2. Results and Discussion

### 2.1. Screening of the Commercial Minerals (Non-Modified)

The screening was conducted in several stages. In the first stage, 3 h-long batch experiments were carried out to remove ammonium ions from the effluents using three natural sorbents, with a particle size of 0.5–1 mm. Their commercial names were: Zeocem Eco, Biozeo R01, and Zeolite Subio.

**Figure 1:**
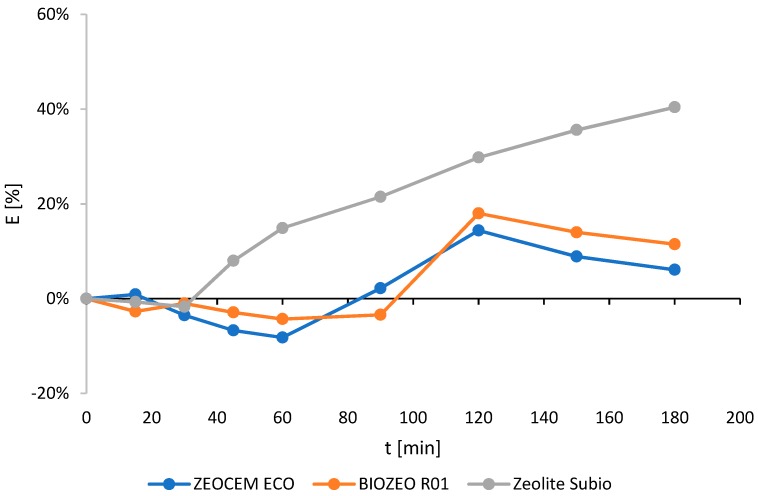
The ammonium ions removal efficiency (E) for Zeocem Eco, Biozeo R01, and Zeolite Subio.

The content of ammonium ions (expressed in %) during the sorption processes is shown in Figure 1. The highest ammonium ions removal efficiency was noted for Zeolite Subio (40.4%), followed by an 18.0% reduction noted for Biozeo R01. It should be noted that in the initial phase, the increase in the concentration of ammonium ions was observed. The increment might have occurred as a result of stirring, and the change in pH, resulting from the addition of sorbent. (Figure 1).

**Figure 2:**
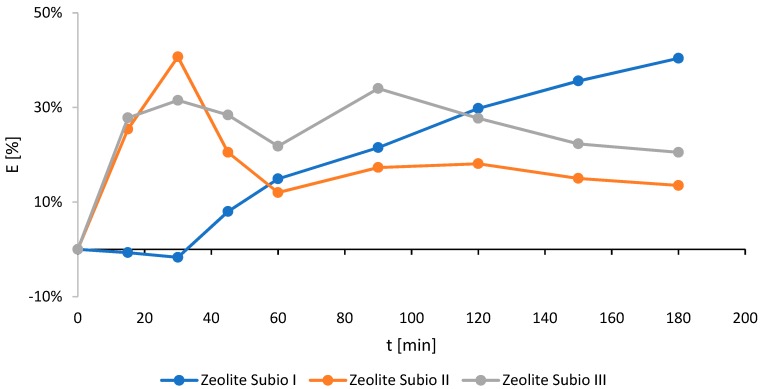
Effect of Zeolite Subio’s particle size on the ammonium ions removal efficiency (E) (particle size I: 0.5–1.0 mm; II: 0.2–0.5 mm; and III: 0.0–0.2 mm).

In regards to zeolite, for which the highest ammonium ions removal efficiency was achieved (Zeolite Subio), a series of experiments was repeated for three different granulometric fractions: I: 0.5–1.0 mm; II: 0.2–0.5 mm; and III: 0.0–0.2 mm. When using fraction II, a similar level of ammonium removal was observed, amounting to approximately 40.5% (Figure 1 and Figure 2). For the smallest sorbent grains (fraction III) the efficiency of 34% was noted (Figure 2).

**Figure 3:**
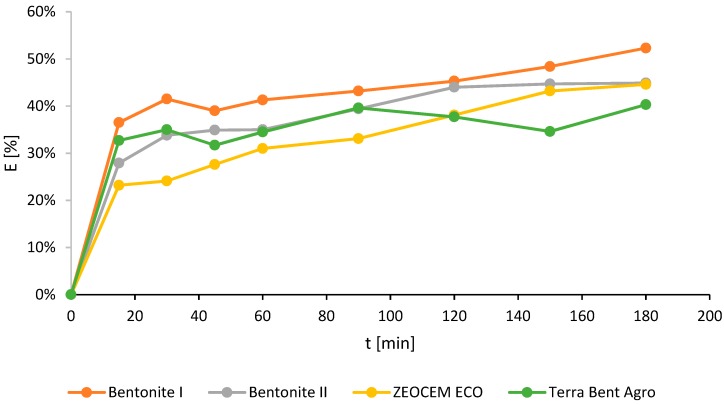
The ammonium ions removal efficiency (E) for Bentonite I and II, Zeocem Eco, and Terra Bent Agro.

The batch experiments were also repeated for non-modified sorbents with the particle size of 0–0.05 mm (Figure 3). The highest removal efficiency of ammonium ions from effluents at 52.3% was observed for the bentonite I sample (by 85% of the montmorillonite content). The lowest efficiency was achieved when Terra Bent Agro (40.3%) was used. For the other two sorbents, the ammonium content decreased from 43.8 to 44.9% (Figure 3).

Alshameri et al. [17] determined the amount of time that is required to remove ammonium from synthetic solutions (NH_4_Cl diluted in distilled water with an initial concentration of ammonium ions 80 mg/L, pH = 7) using natural zeolite. The results confirmed that, due to the prolongation of the process, the ammonium ions removal efficiency increased. The authors obtained 40% removal within the first 10 min, and 78% after 300 min [17]. Similar research was carried out using natural zeolite from Yemen [14]. The removal efficiency of NH_4_^+^ ions at 39% was reported within 10 min, and after 2 h it reached 80%.

The noted NH_4_^+^ adsorption capacity and the maximum ammonium removal efficiencies for the minerals analyzed in this research were illustrated using data presented in Table 1.

The highest adsorption capacity was measured when the finest granulation sorbents were used. The highest ammonium ions removal efficiencies were also noted for them. The removal efficiency for most of the examined minerals reached 40–45% within 180 min (Table 1).

The influence of the duration of the process on the ammonium ions removal efficiency was also examined [15]. The authors used natural zeolite and synthetic sewage, obtained by dilution in distilled water with NH_4_Cl, (NH_4_)_2_HPO_4_, KCl, MgCl_2_, NaCl, CaCl_2_, Na_2_SO_4_, and NaNO_3_. The initial concentration of NH_4_^+^ ions was established at 322 mg/L. The authors stated that along with the prolonged contact between the sewage and the sorbent, the ammonium removal efficiency increased. After 10 min, 42% removal efficiency from synthetic sewage was obtained. However, lengthening the process did not result in further wastewater treatment [15]. The explanation of this phenomenon may be that the most available adsorption sites of zeolite were used, which led to rapid diffusion and equilibrium. When the outer surface is saturated, ion exchange occurs at the pores of the sorbent and its internal surface [10,12]. This may confirm that the ion exchange was the main mechanism of ammonium ions removal, according to the equation (1), proposed by Alshameri et al. [17]:

Zeolite-Na^+^ + NH_4_^+^ = Zeolite-NH_4_^+^ + Na^+^ (1)

During the ion exchange, concentrations of sodium and ammonium ions decrease and increase, this results in the reduction of the driving force of NH_4_^+^ adsorption on zeolites, which may also explain the mechanism that takes place in this method of wastewater treatment [15]. Moreover, the research that has been carried out can confirm the presented statements. The suspensions which occurred in the effluents might have saturated the outer surface of the minerals, and even disturbed the sorption process at the pores of the minerals, which led to a lower efficiency of the effluent treatment.

The treatment of a 1 g/L solution of ammonium chloride using clinoptilolite (of different particle sizes: 20, 30, and 40 mm) was performed by Rahmani et al. [9]. An inverse dependence of the efficiency of the process on the grain size was observed. Using clinoptilolite with a smaller granulation resulted in an improved absorption [9]. Similar findings might be made based on the performed research. The observed ammonium ions removal efficiencies were, in general, at higher levels for minerals with a smaller particle size (Table 1).

### 2.2. The Studies on Activated Sorbent

Taking into consideration the highest adsorption capacity and the maximum removal efficiency obtained in the performed research (Table 1), Bentonite I was chosen to be investigated further, and activated using sodium chloride.

The chemical oxygen demand removal would be an additional benefit for the MBT in the context of internal wastewater treatment. For this reason it was also determined during studies on activated minerals. The highest chemical oxygen demand (COD) removal was obtained for samples activated with a 2.0 mol/L NaCl solution (21.9 %). Due to the other activated samples, the obtained COD removal varied from 10.4 to 11.0 %, and was similar to the COD removal (11.3 %) observed for natural bentonite. The low COD removal may have been influenced by the composition of the effluent.

**Figure 4:**
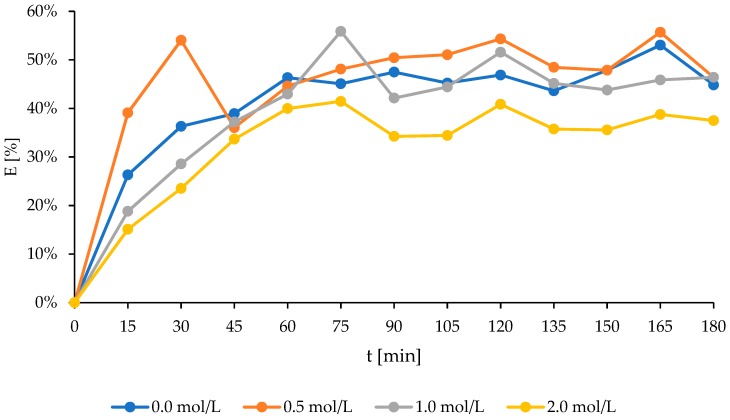
The effect of using different NaCl solutions for the activation of Bentonite I on ammonium ions removal efficiency (E).

Changes in the percentage of the ammonium ions content during the sorption processes were presented in Figure 4. The highest ammonium ions removal efficiency was noted for bentonite activated with a 1mol/L NaCl solution at 55.9 %, after a 55.7% reduction for a 0.5 mol/L activating solution. Due to the use of natural bentonite, an efficiency level that was over 50% was noticed again (Figure 4). Moreover, a 1 mol/L concentration of NaCl was found to be the most effective in pretreatment, resulting in adsorption of NH_4_^+^ ions on Yemeni natural zeolite [14], and Hulaodu natural zeolite [17].

It is essential to use an ion-exchange isotherm to describe the equilibrium relationship between the amount of ion exchanged by a mineral and its equilibrium concentration in the solution [21], which could be used to optimize the use of a mineral as adsorbent [22].

**Figure 5:**
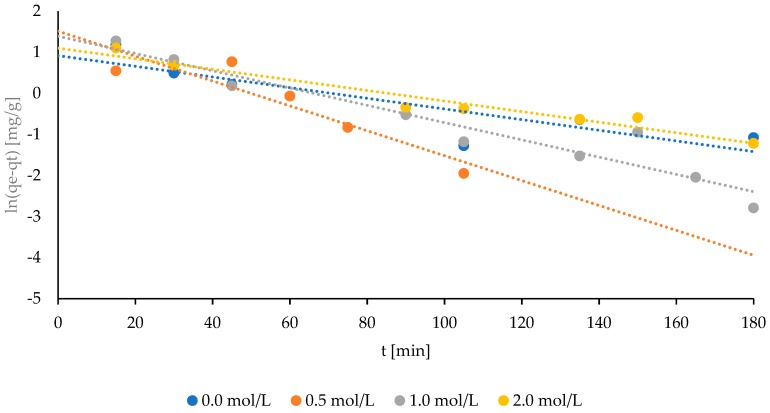
The adaptation of experimental data of ammonium adsorption obtained for the natural and modified Bentonite I–Freundlich isotherms.

The amounts of adsorbed ammonium ions in time using the Freundlich model were corresponded to the experimental values (Figure 5), which indicate the adjustment of data obtained during the experiment to this model. The Freundlich isotherm is applicable to adsorption processes that occur on heterogonous surfaces [23]. This isotherm gives an expression which defines the surface heterogeneity and the exponential distribution of active sites and their energies [24]. Low values of the adsorption process constant (k_1_) were observed, regardless of the NaCl solution used for sorbent activation: 0.013, 0.030, 0.021, and 0.013 min^−1^ for natural bentonite and pretreated bentonite with increasing NaCl concentrations, respectively. Its decrease was observed followed by the increment in the saturation of bentonite with NaCl. A similar pattern was noticed for the adsorption capacity (q_e_).

Kinetic parameters for the ammonium ion exchange using the pseudo-second-order model were shown in Table 2. However, the low values of the correlation coefficient R^2^ can be noticed (Table 2). The highest R^2^ was observed for the bentonite activated by a 2 mol/L sodium chloride solution (0.947), and the lowest for the natural bentonite (0.772) (Table 2). It can be found that the obtained data does not correspond to the Freundlich model, especially for the case of the natural bentonite, and for the bentonite activated with NaCl in relatively low concentrations. The determination coefficient (R^2^) of 0.772 (which means that 77.2% of the total variation is explained by the model) cannot suggest a satisfactory representation of the process model, or a strong correlation between the experimental and predicted values [25,26].

It can be stated that the ion-exchange kinetics of NH_4_^+^ on zeolite were regulated by both surface and intraparticle diffusion processes (Table 1, Table 2, Table 3, and Figure 4). The ammonium exchange process was preceded by surface sorption and intraparticle diffusion. It has been suggested that the first one can be attributed to the instantaneous occupation of the most available surface sites on the zeolites’ particles by the exchanging ammonium ions. The surface of zeolites might be negatively charged thus, making the rate of ion exchange of the NH_4_^+^ very fast. The second region is due to the gradual ion exchange of the NH_4_^+^ into zeolite particles, by intraparticle diffusion through the pores. The resistance to the external mass transfer increases as the intercept increases [14,17].

**Figure 6:**
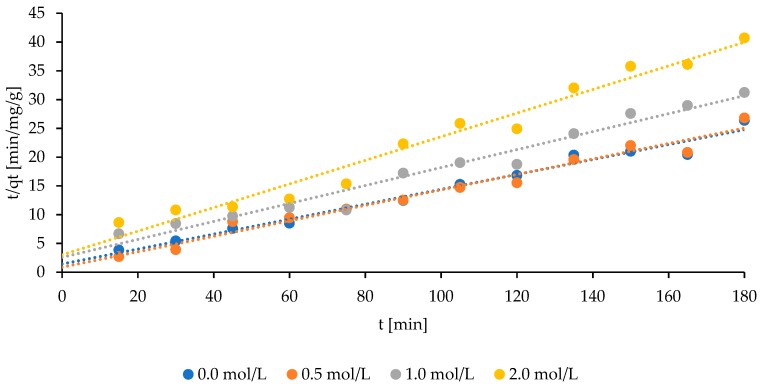
Adaptation of the experimental data of ammonium adsorption obtained for natural and modified Bentonite I—Langmuir isotherm.

A better correlation between the experimental and predicted values was obtained when the Langmuir model was applied (Figure 6).

This statement can be confirmed by the high correlation coefficient, which was at the level of 0.979, 0.972, 0.966, and 0.969 for natural bentonite, and was activated using the increasing NaCl concentrations, respectively (Table 3).

The decrease in the amount of adsorbed ammonium ions is followed by the increment in NaCl bentonite saturation, whereas, the speed ratio k_2_, corresponding with the adsorption, increased with the increment in the saturation of the bentonite with a solution of NaCl. Furthermore, the value of the coefficient h (corresponding to the initial adsorption rate) decreased along with the bentonite saturation increment (Table 3). As it was observed, at the beginning of the runs, an increase in the concentration of ammonium ions caused an increase in the value of the coefficient h (Figure 6, Table 3).

When the Freundlich isotherm was used, the obtained data were not consistent with the data obtained during the experiment (Table 2). The measured kinetic parameters of the adsorption process reached low values, however, it can be observed that the increase in the saturation of bentonite with the NaCl solution had a negative impact on the coefficient k_1_ (Table 2). Based on the noticed correlation coefficient (R^2^), the incompatibility of the Freundlich model can be assumed.

The obtained data were consistent with the experimental data when using the Langmuir isotherm, as evidenced by the correlation coefficient R^2^, exceeding 0.966 (Table 3). With the increase in the saturation of bentonite with the NaCl solution, the amount of adsorbed NH_4_^+^ ions decreased. The Langmuir isotherm accounts for the surface coverage by balancing the relative rates of adsorption and desorption (dynamic equilibrium). Adsorption is proportional to the fraction of the surface of the adsorbent that is open, while desorption is proportional to the fraction of the adsorbent surface that is covered [27].

The k_2_ factor increased along with the increasing saturation of bentonite. The adsorption of leachate pollutants with ammonium ions was consistent with the Langmuir model. Vázquez et al. [28] also fitted the Langmuir model, and achieved the highest amount of removed ammonium ions (38.12 mg/g) using granular activated carbon. Also, Alshameri et al. [14] obtained much higher R^2^ values (0.997) for the Langmuir model, compared to the Freundlich model (0.736) for the ammonium removal by natural zeolite. However, Huang et al. [15] noticed that in the course of removing ammonium from swine wastewater using zeolite, similar R^2^ levels were obtained (Langmuir and Freundlich models: 0.997 and 0.985, respectively). It can be assumed that at least two mentioned models should be investigated and compared to verify the correlation between the experimental and predicted values.

## 3. Materials and Methods

### 3.1. The Commercial Minerals

The minerals used in the present study belong to the silicate family with commercial names: Zeocem Eco provided by Zeocem a.s., Bystré, Slovakia with particle sizes of 0–0.05 mm and 0.5–1 mm, Biozeo R01 from BioDrain Ltd., Rzeszów, Poland with a 0.5–1.0 mm fraction, Zeolite Subio provided by Subio Eko Polska Ltd., Krzyżanowice, Poland with a granulation of 0–0.2 mm, 0.2–0.5 mm, and 0.5–1.0 mm, Bentonite I and II provided by Certech Ltd., Niedomice, Poland with a grain size of 0–0.05 mm, and Terra Bent Agro provided by Celpap Ltd., Wieliczka, Poland, with a granulation of 0–0.05 mm.). The mineral and chemical composition of the used minerals, based on the product data sheets, are presented in Table 4.

### 3.2. The Industrial Wastewater

The industrial wastewater used in this study consisted of mixed effluents from both biological treatment processes (anaerobic digestion and oxygen stabilization) of the organic fraction of municipal solid waste, that were performed in the mechanical-biological treatment (MBT) plant (ZGO Gać), and were collected in a 650 m^3^ tank and used in the research. Its characteristics can be found in Table 5. All of the analyses have been performed using APHA [29].

### 3.3. Ammonium ion Exchange

Batch ion-exchange experiments were carried out using flasks (250 mL) in a shaker and shaken at 120 rpm at room temperature in the course of 3 h using the mineral/liquid ratio of 10 g/100 mL. The pH value of the effluents was not corrected. The suspended solids were not removed. Every 15 m the electrode was introduced to measure the content of the ammonium ions.

The screening of minerals with a particle size of 0.5–1.0 mm was conducted (Zeocem Eco, Biozeo R01, Zeolite Subio) in the first stage of the ammonium ion-exchange research. For the mineral with the highest removal efficiency the experiment was repeated using its different particle sizes (Zeolite Subio). Furthermore, the 3rd trial was performed using sorbent with the smallest granulation (Bentonite I and II, Zeocem Eco, and Terra Bent Agro). Among all of them, the sorbent with the highest ions removal efficiency was chosen for the activation (Bentonite I).

### 3.4. The Sorbent Activation

The activation process was carried out by mixing natural sorbent (Bentonite I) with an aqueous solution of sodium chloride (POCh, Gliwice, Poland). To examine the effect of various sodium concentrations, different NaCl concentrations were used (0.5, 1.0, and 2.0 mol/L). The suspension of 25 g samples of bentonite and a 250 mL sodium chloride solution were stirred in the flasks (500 mL), and kept in water batch (80 °C) for 1 h. Subsequently, the suspension was filtered and washed with distilled water. Afterwards, the wet material was dried at 100 °C for 24 h.

### 3.5. Analytical Methods

The NH_4_^+^ adsorption capacity (q) and removal efficiency (E) of mineral were calculated using the equations:

q = (C_0_ − C_k_) × (V/m) (2)

and

E = [(C_0_ − C_e_)/C_0_] × 100% (3)

respectively, where: q (adsorption capacity at time t) is the total amount of adsorbed NH_4_^+^ ions per unit of weight of mineral at time t (mgN/g_z_); C_0_,C_t_, C_e_ are the initial, time t, and equilibrium concentrations of NH_4_^+^ in solution (mg N/L); V is the initial volume of treated effluent (L); m is the adsorbent mass (g).

The content of ammonium ions in the wastewater was determined using the ammonium ion-selective electrode (DETEKTOR, Raszyn, Poland) with the silver-chloride reference electrode (DETEKTOR, Raszyn, Poland) and the ionometer (ELMETRON CPI-505, Zabrze, Poland). To control the values obtained by means of using the ammonium ion-selective electrode, the spectrophotometric Hach-Lange cuvette tests (LCK 302) (HACH LANGE Ltd., Wrocław, Poland) [30] have been used, and the initial and final concentration has been measured in the course of the experiments.

The chemical oxygen demand (COD) was determined spectrophotometrically using the HACH-Lange cuvette tests (LCK 514) (HACH LANGE Ltd., Wrocław, Poland) [30] at the beginning and end of each experiment, when the sorbent activation with NaCl has been studied to verify the possibility of COD removal.

### 3.6. Ammonium Ion-Exchange Isotherms

Information pertaining to adsorption equilibrium is the most important information that is required for the proper understanding of an adsorption process [31]. The profile obtained from the study of ammonium removal using mineral sorbent was used to obtain adsorption isotherms by using well-known equations of the Langmuir and Freundlich models [15,31].

## 4. Conclusions

The present research examined various types of natural sorbents, with different granulations, determining the efficiency of ammonium ions removal from the waste treatment effluents and the impact of the processing time. The highest adsorption capacity (4.92 mg/g) with the maximal removal efficiency (52.3%) was obtained for Bentonite I, with the particle size of 0–0.05 mm. Generally, it was found that the lower the mineral size, the higher the adsorption capacity as well as removal efficiency.

The chosen bentonite sample was pretreated with various NaCl solutions (0.5, 1.0, and 2.0 mol/L). The activated mineral showed higher removal efficiency compared to natural bentonite. Maximum efficiency (55.7%) was measured for bentonite I pretreated with the 1 mol/L NaCl solution. Regarding the sample activated using the 2 mol/L, the concentration removal efficiency (41.4%) was lower than natural mineral efficiency.

The appropriate modeling and interpretation of adsorption isotherms affect the level of data accuracy obtained in adsorption processes. The Langmuir adsorption isotherm corresponds well with the equilibrium adsorption data (R^2^ ranged 0.97–0.98), while the Freundlich model was found to be incompatible (R^2^ at 0.77 level).

The obtained results confirmed the observations of the research carried out by other authors. However, the solutions tested by other researchers were free of suspensions that could potentially limit the effectiveness of treatment. The suspensions in wastewater can also hinder their purification from ammonium ions by means of adsorption. For this reason, most research on this process described in the literature was carried out on synthetically prepared solutions, while our research was performed using the untreated industrial wastewater. Thus, the novel method for wastewater treatment was applied in the MBT plant.

## Figures and Tables

**Table 1:** Effect of using different sorbents on the NH_4_^+^ adsorption capacity (q) and maximum removal efficiency (E) obtained in performed research.

Mineral Commercial Name	Particular Size [mm]	Adsorption Capacity (q) [mgN/g_z_]	Maximal Removal Efficiency (E) [%]	Needed Contact Time to Achieve Maximum E [min]
Zeocem Eco	0.5–1	0.39	14.4	120
Biozeo R.01	0.5–1	0.77	18.0	120
Zeolite Subio I	0.5–1	3.05	40.4	180
Zeolite Subio II	0.2–0.5	0.99	40.7	30
Zeolite Subio III	0–0.2	1.43	34.0	90
Bentonite I	0–0.05	4.92	52.3	180
Bentonite II	0–0.05	4.22	44.9	180
Zeocem Eco	0–0.05	4.2	44.6	180
Terra Bent Agro	0–0.05	3.79	40.3	180

**Table 2:** The kinetic parameters for ammonium ion exchange using the Freundlich model (0.5 M, 1 M, and 2 M indicate NaCl concentrations of 0.5, 1.0, and 2.0 mol/L respectively).

Mineral	k_1_ [min^−1^]	q_e_ [mgN/g_z_]	R^2^
Natural bentonite I	0.013	7.164	0.772
0.5 M activated bentonite I	0.030	7.285	0.840
1 M activated bentonite I	0.021	5.828	0.924
2 M activated bentonite I	0.013	4.748	0.947

**Table 3:** The kinetic parameters for ammonium ion exchange using the Langmuir model (0.5 M, 1 M, and 2 M indicate NaCl concentrations of 0.5, 1.0, and 2.0 mol/L, respectively).

Mineral	k_2_ [g/mg·min]	q_e_ [mgN/g_z_]	h [mg/g·min]	R^2^
Natural Bentonite I	0.013	7.164	6.657	0.979
0.5 M activated Bentonite I	0.134	7.285	7.127	0.972
1 M activated Bentonite I	0.156	5.828	5.298	0.966
2 M activated Bentonite I	0.205	4.748	4.626	0.969

**Table 4:** The mineral and chemical composition of used sorbents.

Mineral and Chemical Composition [%]	Zeocem Eco	Zeolite Subio	Bentonite I	Bentonite II	Terra Bent Agro	Biozeo R01
Clinoptilolite	84	84				60
Cristobalite	8	8				
Clayish mica	4	4				
Plagioclase	3–4	3–4				
Edisonite	0.1–0.3	0.1–0.3				
Montmorillonite			85	65	65	
SiO_2_	65–71.3	65–71.3	70–80	60–65	60–80	70.6
Al_2_O_3_	11.5–13.1	11.5–13.1	13–17	15–20	11–20	12.32
Fe_2_O_3_	0.7–1.9	0.7–1.9	1–2	5–7	ND	1.48
CaO	2.7–5.2	2.7–5.2	0.5–1.5	2–4	1.5–5.2	3.42
TiO_2_	0.1–0.3	0.1–0.3	0.05–0.15	0.5–1.0	ND	0.71
MgO	0.6–1.2	0.6–1.2	0.8–1.8	1–2	ND	0.96
MnO	ND	ND	0.05	<0.05	ND	0.02
K_2_O	2.2–3.4	2.2–3.4	0.5–2.0	0.5–1.0	0.5–3.4	2.83
Na_2_O	0.2–1.3	0.2–1.3	<0.01	<0.01	ND	0.68
P_2_O_5_	ND	ND	<0,01	<0,1	ND	ND
ZrO_2_	ND	ND	<0.01	<0.01	ND	ND
Cr_2_O_3_	ND	ND	<0.01	<0.02	ND	ND
SO_3_	ND	ND	<0.01	<0.01	ND	ND
Si/Al.	4.8–5.4	4.8–5.4	ND	ND	ND	ND

Before each run, the minerals were dried in an oven at 100 °C for 1 h.

**Table 5:** The industrial wastewater characterization.

Parameter	Value
pH [-]	7.8 ± 0.3
Chemical Oxygen Demand (COD) [g/L]	18.7 ± 1.3
Biological Oxygen Demand (BOD) [g/L]	6.0 ± 0.5
Ammonium nitrogen [g/L]	0.8 ± 0.2
Total nitrogen [g/L]	1.1 ± 0.3
Total Organic Carbon (TOC) [g/L]	3.1 ± 0.2
Total suspended solids (TSS) [g/L]	0.6 ± 0.2
Cl^−^ [g/L]	1.3 ± 0.2
